# A revision of infrageneric classification in *Astelia* Banks & Sol. ex R.Br. (Asteliaceae)

**DOI:** 10.3897/phytokeys.52.4768

**Published:** 2015-07-13

**Authors:** Joanne L. Birch

**Affiliations:** 1 Department of Botany, University of Hawaii at Manoa, Honolulu, HI, 96822, USA; 2 Present address: Royal Botanic Gardens Victoria, Birdwood Avenue, Melbourne, Victoria 3004, Australia

**Keywords:** Asparagales, Asteliaceae, Austral, Australia, dioecy, New Zealand, Pacific

## Abstract

Systematic investigations and phylogenetic analyses have indicated that *Astelia*, as currently circumscribed, is paraphyletic, with *Collospermum* nested within it. Further, Astelia subgenus Astelia is polyphyletic, and *Astelia* subgenera *Asteliopsis* and *Tricella* are paraphyletic, as currently circumscribed. Revision of the subgeneric classification of *Astelia* is warranted to ensure classification accurately reflects the evolutionary history of these taxa. *Collospermum* is relegated to synonymy within *Astelia*. *Astelia* is dioecious or polygamodioecious, with a superior ovary, anthers dorsi- or basifixed, pistillodes or pistils that have a single short or poorly defined style, a 3 lobed stigma, and fleshy uni- or trilocular fruit with funicular hairs that are poorly to well developed. Astelia subgenus Collospermum (Skottsb.) Birch is described. A key to *Astelia* sections is provided. *Astelia hastata* Colenso, *Astelia montana* Seem., and *Astelia microsperma* Colenso *pro parte* are resurrected and the new combination *Astelia samoense* (Skottsb.) Birch, **comb. nov.** is made.

## Introduction

*Astelia* Banks & Sol. ex R.Br. is the largest genus in Asteliaceae Dumort., containing twenty-six species and three non-nominotypical varieties with an Austral-Pacific distribution. *Astelia* species exhibit a range of growth forms including low, cushion-forming and tall, clustered habits. *Astelia* species grow in a diverse range of habitats including coastal, lowland wetlands, tropical and temperate lowland forests, tropical montane cloud forests, sub-alpine heath, alpine fellfields and grasslands, and bog habitats. Many *Astelia* species are facultatively epiphytic and three species are primarily epiphytic. *Collospermum* Skottsb. includes four species that occur in lowland forests and in lowland and tropical montane cloud forests in New Zealand, the Independent State of Samoa and the Republics of Fiji and Vanuatu. All species exhibit a tall, clustered habit and are primarily epiphytic, although plants that fall to the ground can persist for long periods.

*Astelia* and *Collospermum* share many morphological characters (Bayer et al. 1998, Rudall et al. 1998). All Asteliaceae genera have branched hairs that are otherwise uncommon in the Lilianae (Bayer et al. 1998) and those of *Astelia* and *Collospermum* are dense, at least in the young leaves of most species. The tomentum of *Astelia* and *Collospermum* consist of a multi-celled stalk, frayed unicellular filaments that form a basal wool adjacent to the stalk, and linear or ovate scales that are a single cell thick (McCarthy 1928, Skottsberg 1934, L. B. Moore 1980, Rudall et al. 1998). In *Astelia* the scales are linear, may be short or long, and the stalk is attached at the base of the scale. In *Collospermum*, the scales are ovate, short, and the stalk is peltate. Scales may be present on the adaxial and/or abaxial leaf surfaces, inflorescences, and flowers. On the leaves of *Astelia* species the scales may become fused to form a membranous pellicle over the leaf epidermis. *Astelia* and *Collospermum* have superior ovaries that are uni- or trilocular and trilocular, respectively. Published chromosome numbers for *Astelia* taxa range from 2*n* = 60 to 2*n* = 210 (Wheeler 1966, Dawson and Beuzenberg 2000, de Lange et al. 2004). Darlington and Wylie (1955) proposed a basic chromosome number for *Astelia* of *x* = 8, but Wheeler (1966) considered the basic chromosome number to be *x* = 5, 7, or 35. *Collospermum hastatum* and *Collospermum microspermum* are the only *Collospermum* taxa for which chromosome numbers are known and both have a chromosome number of *n* = 35.

Skottsberg (1934) segregated *Collospermum* from *Astelia* based on the presence of simple lateral racemes, dimorphism of staminate and pistillate plants, basifixed anthers, long style papillae, and mucilaginous seed hairs of the former, which differ from the predominantly paniculate lateral racemes, versatile anthers, and poorly developed mucilaginous seed hairs of the latter (Birch unpublished PhD thesis 2011, Birch et al. 2012). However, morphological (Bayer et al. 1998, Birch unpublished PhD thesis 2011), cytological (Wheeler 1966, Moore 1980), and molecular (Birch unpublished PhD thesis 2011, Birch et al. 2012) data suggested a close evolutionary relationship between these genera. Moore (1980) considered that a re-evaluation of the circumscriptions of *Astelia* and *Collospermum* was warranted due to the production of viable progeny from intergeneric crosses. *Collospermum*, while monophyletic, was nested within *Astelia* in phylogenetic reconstructions based on combined chloroplast and nuclear sequence data applying Bayesian inference, maximum likelihood, and maximum parsimony criteria (Birch unpublished PhD thesis 2011, Birch et al. 2012). As a result, those authors recognized a broadly circumscribed *Astelia* *s.l.*, including *Collospermum*.

Within *Astelia*, Skottsberg (1934) recognized three subgenera (Astelia subg. Astelia Skottsb., Astelia subg. Asteliopsis Skottsb., and Astelia subg. Tricella Skottsb.) based on open or cushion-forming growth form, degree of tepal fusion, ovary division, and seed shape. Within these subgenera, he recognized seven sections (Astelia sect. Astelia Skottsb., Astelia sect. Desmoneuron Skottsb., Astelia sect. Isoneuron Skottsb., Astelia sect. Micrastelia Skottsb., Astelia sect. Palaeastelia Skottsb., Astelia sect. Periastelia Skottsb., and Astelia sect. Tricella Skottsb.) based on leaf venation, pistillode size, seed surface features, and extent of funicle development. In phylogenetic analyses (Birch unpublished PhD thesis 2011, Birch et al. 2012), each of Skottsberg’s (1934) sections, except Astelia sect. Tricella, were monophyletic, Astelia subg. Astelia and Astelia subg. Asteliopsis were polyphyletic, and Astelia subg. Tricella was paraphyletic. A revised circumscription of *Astelia* subgenera is proposed that accurately reflects the evolutionary relationships within the genus. *Collospermum* is relegated to synonomy under *Astelia*. Skottsberg’s sections are retained as they are monophyletic and accurately capture the extensive morphological diversity that is present within the subgenera.

A revision based on recognition of monophyletic taxa is proposed here. Multiple characters support the proposed circumscription of *Astelia*. All taxa are dioecious or polygamodioecious, with a superior ovary, dorsi- or basifixed anthers, pistillodes or pistils that have a single short or poorly defined style, a 3 lobed stigma, and fleshy uni- or trilocular fruit with funicular hairs that are poorly to well developed.

## Methods

### Taxonomic sampling

All *Astelia* taxa, (twenty-six species and three non-nominotypical varieties) and all *Collospermum* (four species) were included in this study. Herbarium specimens were examined from the following herbaria: Auckland War Memorial Museum (AK), Herbarium Pacificum (BISH), Allan Herbarium (CHR), Harvard University (GH), Kew Royal Botanic Gardens (K), National Herbarium of Victoria (MEL), Missouri Botanical Garden (MO), Herbier National de Paris (P), National Tropical Botanical Garden (PTBG), United States National Herbarium (US), and Museum of New Zealand Te Papa Tongarewa (WELT). Type specimens were examined from AK, BISH, MEL, P, WELT and digital images of type specimens were examined from CHR and K (Herbarium abbreviations follow Index Herbariorum (Thiers continuously updated).

### Morphological data and analyses

Morphological data were obtained for 410 herbarium specimens (Appendix 1). Data were obtained for ten specimens per species, including five staminate and five pistillate specimens for species with unisexual flowers. Measurements and scores were averaged across all specimens to give a mean value for each taxon. Flower and fruit color data were obtained from multiple sources including field observations, specimen label data, and taxon descriptions in national floras (Drake del Castillo 1893, L. B. Moore and Edgar 1976, Coode 1978, D. M. Moore 1983, Williams 1987, Wagner et al. 1999).

Morphological characters that varied at or below the genus rank were measured or scored for all *Astelia* and *Collospermum* taxa in the field and/or herbarium. Herbarium specimens were studied under a dissecting microscope and measurements obtained using digital calipers. Pollen and seed characters were examined directly from material obtained from herbarium specimens after coating with gold-palladium using a Hitachi S-4800 field emission scanning electron microscope (SEM) at the Biological Electron Microscope Facility, Pacific Biosciences Research Center of the University of Hawai‘i at Mānoa. Images were digitally processed and the final plates were prepared in Photoshop 10.0.

**Figure 1.**
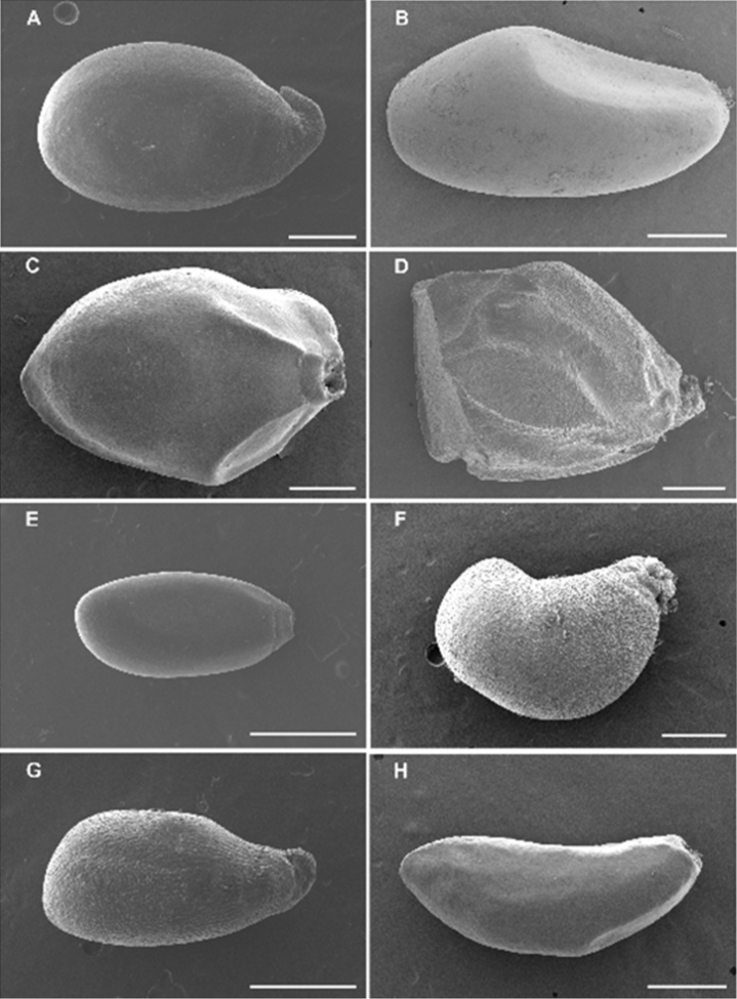
Scanning electron micrographs of *Astelia* seeds showing size, shape, and funicle characters. **A** Astelia (sect. Astelia) linearis var. linearis; ovoid, funicle long **B** Astelia (sect. Tricella) petriei; ovoid, funicle short **C** Astelia (sect. Tricella) chathamica; ovoid, funicle short **D** Astelia (sect. Isoneuron) banksii: polygonal-turbinate, funicle ribbed **E** Astelia (sect. Micrastelia) pumila; ovoid, funicle short **F** Astelia (sect. Desmoneuron) solandri; obovoid-reniform, funicle ribbed **G** Astelia (sect. Isoneuron) neocaledonica; obovoid, funicle ribbed **H** Astelia (sect. Tricella) menziesiana; fusiform, funicle short. Scale bars = 0.5 mm. SEM images created by J.L. Birch.

## Taxonomic treatment

### Astelia

Taxon classificationPlantaeAsparagalesAsteliaceae

Banks & Sol. ex R.Br.

Astelia Banks & Sol. ex R.Br., Prodr. 291. 1810. *nomen conservandum* (International Botanical Congress and JH Wiersema 2015). Type: *Astelia alpina* R.Br.
Funckia Willd., Mag. Neuesten Entdeck. Gesammten Naturk. Ges. Naturf. Freunde Berlin 2: 19. 1808, *nomen rejiciendum*. Type: *Funckia magellanica* Willdenow, *nomen illegitimum* (*Melanthium pumilum* G. Forster)
Hamelinia A.Rich., Voy. Astrolabe 1: 158. 1832. Type: *Hamelinia veratroides* A.Rich.

#### Note.

Herbaceous perennials, terrestrial or epiphytic, often growing in clusters with three ramets in trigonal arrangement, some species turf-forming, rhizomatous, dioecious or polygamodioecious. Leaves: 3-ranked, linear, ensiform, or subulate; leaves usually keeled, margins erect or revolute; leaf sheath closed, with surface obscured by dense long white hairs; parallel venation, variously incrassate; tomentum composed of scales and lanate wool at base of scale stalk, scales with basal stalk or peltate. Inflorescence: a terminal panicle, sometimes reduced to a few flowers; lateral branches racemes or sub-panicles, subtended by foliaceous or membranous, linear or lanceolate spathes; peduncle tomentum composed of distinct, narrow scales with dense basal wool. Flowers: pedicillate; bracts membranous, linear or spathulate; perianth membranous or fleshy, 6 tepals in 2 series; connate at base into tube of variable length; outer tepals triangular to lanceolate, with three veins, scales present over entire surface; inner tepals linear with one midvein, scales present along midvein only. Staminate flowers: lobes recurved; stamens 6; filaments filamentous, adnate to tepals at base of tepal lobes; anthers elliptic or linear-hastate, dorsifixed and versatile or basifixed and immobile, latrorse; pistillode present, style undifferentiated or distinct; stigma not formed. Pistillate flowers: 6 reduced staminodes present, adnate to base of tepal lobes, filament filamentous, anthers flattened, sterile; ovary superior, uni- or trilocular, placentation parietal from three placentas or axile, with subapical placentas, ovules few to many; style distinct or undifferentiated, stigmas 3. Fruit: berry, stigma typically persistent. Seeds: black, obovoid, ellipsoid, fusiform, or polygonal; testa smooth or sculptured; funicle with mucilaginous funicular hairs poorly or well developed, funicle hairs surrounding the seeds and either adhering to the testa or not.

### Astelia Banks & Sol. ex R.Br. subg. Astelia

Taxon classificationPlantaeAsparagalesAsteliaceae

#### Note.

Flowers with a very short perianth tube (0.1–0.8 mm); anthers dorsifixed, versatile; ovary unilocular; seeds ovoid; funicle long, curved, with mucilaginous funicular hairs poorly developed that surround but do not adhere to the seed.

#### Remarks.

Molecular (Birch et al. 2012) and morphology-based phylogenetic analyses (Birch unpublished PhD thesis 2011) indicate that Astelia subg. Astelia, as circumscribed by Skottsberg (1934), is polyphyletic. Astelia subg. Astelia is revised to include Astelia sect. Palaeastelia and Astelia sect. Astelia, which form a clade. Astelia sect. Desmoneuron is placed in a different clade and it is excluded from Astelia subg. Astelia.

### Astelia sect. Astelia

Taxon classificationPlantaeAsparagalesAsteliaceae

#### Note.

Low, compact, growth form (including cushion or turf forming taxa); leaves, linear, ensiform, or subulate; reduced inflorescences bearing few flowers; staminate flowers with short filaments (0.5–1.6 mm); pistillate flowers with long outer tepals (4.5–7.0 mm); ovary unilocular, long (4.3–8.3 mm); fruit ovoid or oblong; few or many seeds per fruit (< 25), seeds short and narrow (1.1–2.0 × 0.5–1.3 mm).

#### Included taxa and distribution.

Australia Astelia alpina R.Br. var. alpina, Astelia alpina var. novae-hollandiae Skottsb. Indonesia (Papua Province), Papua New Guinea *Astelia papuana* Skottsb.. New Zealand Astelia linearis Hook.f. var. linearis, Astelia linearis var. novae-zelandiae Skottsb., *Astelia subulata* Cheeseman

#### Habitat.

Lowland (low latitudes) to sub-alpine (mid and higher latitudes) herb-fields particularly on wet substrates (seeps, swamps etc.).

### Astelia sect. Palaeastelia

Taxon classificationPlantaeAsparagalesAsteliaceae

Skottsb.

Astelia sect. Palaeastelia Skottsb., Kongl. Svenska Vetenskapsakad. Handl. ser. 3, 14(2): 24. 1934.

#### Note.

Open growth form; leaves linear; large inflorescences bearing many flowers; staminate flowers with intermediate length filaments (1.7–4.6 mm); pistillate flowers with short outer tepals (2.3–4.4 mm); ovary unilocular, intermediate length (3.2–4.2 mm); fruit ovoid, many seeds per fruit (< 20); seed intermediate length and narrow (2.4–2.9 × 1.2–1.3 mm). Type: *Astelia hemichrysa* (Lam.) Kunth.

#### Included species and distribution.

Mascarene Islands, Réunion Island. *Astelia hemichrysa* (Lam.) Kunth

#### Habitat.

Tropical forest.

### Astelia subg. Asteliopsis

Taxon classificationPlantaeAsparagalesAsteliaceae

Skottsb.

Astelia subg. Asteliopsis Skottsb., Kongl. Svenska Vetenskapsakad. Handl. ser. 3, 14(2): 46. 1934.

#### Note.

Flowers with a short perianth tube (0.2–1.1 mm); anthers dorsifixed, versatile; ovary uni- (Astelia sect. Desmoneuron) or trilocular (Astelia sect. Isoneuron); seeds obovoid, obovoid-reniform, or turbinate-polygonal; funicle ribbed, mucilaginous funicular hairs well developed that surround but do not adhere to the seed; Type: *Astelia trinervia* Kirk, designated here.

#### Remarks.

Phylogenetic analyses indicate that Astelia subg. Asteliopsis, as circumscribed by Skottsberg (1934), is polyphyletic (Birch unpublished PhD thesis 2011, Birch et al. 2012). Astelia subg. Asteliopsis is revised to include Astelia sect. Isoneuron and Astelia sect. Desmoneuron, which form a clade. But, as Astelia sect. Periastelia, is placed in a different clade, it is excluded from Astelia subg. Asteliopsis.

### Astelia sect. Desmoneuron

Taxon classificationPlantaeAsparagalesAsteliaceae

Skottsb.

Astelia sect. Desmoneuron Skottsb., Kongl. Svenska Vetenskapsakad. Handl. ser. 3, 14(2): 34. 1934.

#### Note.

Open growth form; leaves linear with a group of three subequal lateral nerves conspicuous in lower half; inflorescences bearing many flowers; staminate flowers with short filaments (0.6–2.1 mm); pistillate flowers with short outer tepals (1.9–4.2 × 0.7–1.8 mm); ovary unilocular, short (1.3–2.8 mm); fruit ampulliform, many seeds per fruit (8–32); seeds short and narrow (1.4–1.8 × 0.5–1.0 mm). Type: *Astelia trinervia* Kirk, designated here.

#### Included species and distribution.

New Zealand: *Astelia solandri* A.Cunn., *Astelia trinervia* Kirk. Society Islands, Tahiti: *Astelia nadeaudii* Drake & F.Br.

#### Habitat.

Temperate forest (*Astelia solandri* and *Astelia trinervia*) and tropical montane cloud forest (*Astelia nadeaudii*).

### Astelia sect. Isoneuron

Taxon classificationPlantaeAsparagalesAsteliaceae

Skottsb.

Astelia sect. Isoneuron Skottsb., Kongl. Svenska Vetenskapsakad. Handl. ser. 3, 14(2): 51. 1934.

#### Note.

Open growth form; leaves linear; inflorescences bearing many flowers; staminate flowers with short filaments (0.6–1.9 mm); pistillate flowers with short outer tepals (2.7–4.0 × 0.7–2.0 mm); ovary trilocular, intermediate length (2.2–4.4 mm); fruit ovoid, many seeds per fruit (11–18); seeds small and narrow (1.3–2.2 × 0.8–1.4 mm). Type: *Astelia banksii* A.Cunn., designated here.

#### Included species and distribution.

New Caledonia: *Astelia banksii* A.Cunn., *Astelia neocaledonica* Schltr.

#### Habitat.

Lowland coastal cliffs (*Astelia banksii*) and lowland tropical forest (*Astelia neocaledonica*).

### Astelia subg. Collospermum

Taxon classificationPlantaeAsparagalesAsteliaceae

(Skottsb.) Birch stat. nov.

urn:lsid:ipni.org:names:77148154-1

Basionym: Collospermum Skottsb., Kongl. Svenska Vetensk. Acad. Handl. Ser. 3, 14(2): 72. 1934. Type: *Collospermum hastatum* (Colenso) Skottsb. [Lectotypified by Moore and Edgar (1976)].

#### Note.

Open growth form; leaves linear or ensiform with dark coloration at base and peltate branched hairs; inflorescences bearing many flowers; flowers with a long perianth tube (1.3–3.1 mm); staminate flowers with long filaments (3.8–8.6 mm), anthers basifixed, immobile; pistillate flowers with long outer tepals (1.5–6.2 × 0.9–1.6 mm); ovary trilocular, intermediate length (2.1–4.8 mm); fruit globose or obpyriform, with few to many seeds per fruit (1–22); seeds ellipsoid or ovoid, small and narrow (1.3–2.1 × 0.1–1.1 mm); funicle short, truncate, with well-developed mucilaginous funicular hairs that adhere to the seed.

#### Remarks.

Species published under *Collospermum* must be transferred as *Astelia* has nomenclatural priority. Synapomorphies recognized for the genus *Collospermum* (Skottsberg 1934) remain valid for Astelia subg. Collospermum.

#### Included species and distribution.

New Zealand: *Astelia hastata* Colenso, *Astelia microsperma* Colenso *pro parte*. Republic of Fiji, Viti Levu, Vanua Levu, Kandavu; Republic of Vanuatu, Espiritu Santo, Tanna, Aneityum: *Astelia montana* Seem. Independent State of Samoa, Savai’i, Upolu: *Astelia samoense* (Skottsb.) Birch.

#### Habitat.

Lowland temperate forest (*Astelia hastata* and *Astelia microsperma*) and montane tropical cloud forest (*Astelia montana* and *Astelia samoense*).

### Astelia hastata

Taxon classificationPlantaeAsparagalesAsteliaceae

Colenso

Astelia hastata Colenso, Trans. & Proc. New Zealand Inst. 19. 265. 1887.
Funckia hastata Kuntze, Revis. Gen. Pl. 2: 711. 1891, *nomen rejiciendum*.
Astelia furfuracea Banks et Solander MSS, *fide* C. Skottsberg, Kongl. Svenska Vetensk. Acad. Handl. Ser. 3, 14(2): 77. 1934.
Collospermum hastatum (Colenso) Skottsb., Kongl. Svenska Vetensk. Acad. Handl. Ser. 3, 14(2): 77. 1934; based on *Astelia hastata* Colenso.

#### Type.

NEW ZEALAND. North Island. Hilly country north of Napier, County of Wairoa. January 1886, *A. Hamilton s.n.* (Lectotype: K [000524883, digital image!], staminate, designated by Skottsberg, 1934, 79; Isolectotypes: K [000524884, digital image!], staminate, pistillate; AK [3191!], staminate, pistillate).

### Astelia microsperma

Taxon classificationPlantaeAsparagalesAsteliaceae

Colenso pro parte

Astelia microsperma Colenso *pro parte*, Trans. & Proc. New Zealand Inst. 17: 251. 1885 (description of fruit only). Type: NEW ZEALAND. North Island. Seventy-mile Bush, between Norsewood and Danniverke, County of Waipawa. 1884, *W. Colenso s.n.* (Lectotype: K [000524879, digital image!], fruiting material in packet, designated by Skottsberg, 1934, 81).
Collospermum microspermum Skottsb., Kongl. Svenska Vetensk. Acad. Handl. Ser. 3, 14(2): 82. 1934; based on *Astelia microsperma* Colenso.
Funckia microsperma Kuntze, Revis. Gen. Pl. 2: 711. 1891, *nomen rejiciendum*.
Astelia albicans Colenso, Trans. & Proc. New Zealand Inst. 17: 252. 1885. Type: NEW ZEALAND. North Island. East slopes of Ruahine mountain range, county of Waipawa. Jan. 1884, *A. Hamilton s.n.* (Syntype: K [000524880, digital image!], staminate, pistillate). Skottsberg (1934, 88) identified a single specimen at K as the type material and the specimen in his plate 20 (K000524880) is consistent with the specimen described as the type. As *Astelia* is dioecious, the inclusion of one staminate and one pistillate inflorescence on the specimen means that it represents two distinct collections and each is rendered a syntype.
Funckia albicans Kuntze, Revis. Gen. Pl. 2: 711. 1891, *nomen rejiciendum*.
Astelia graminifolia Colenso, Trans. & Proc. New Zealand Inst. 19: 267. 1887.
Astelia microsperma Type: NEW ZEALAND. North Island. Woods, hilly country north of Napier, County of Wairoa, 1886, *A. Hamilton s.n.* (Lectotype: K [000524881, digital image!], pistillate, designated by Skottsberg, 1934, 85).
Funckia graminifolia Kuntze, Revis. Gen. Pl. 2: 711. 1891, *nomen rejiciendum*.
Astelia planifolia Colenso Trans. & Proc. New Zealand Inst. 20: 209-210. 1888.
Astelia microsperma Type: NEW ZEALAND. North Island, Pohue, hilly country west of Napier, Hawke’s Bay. 1884. *A. Hamilton s.n.*; no specimens located.

#### Remarks.

*Astelia microsperma* was described by Colenso based on a specimen at Kew that contained material from two species (Skottsberg 1934). The species description “referred to both, but mainly to the pistillate raceme in the envelope” (Skottsberg 1934, 82). Skottsberg (1934) lectotypified the fruiting material in the packet (K000524879) as the type material of *Astelia microsperma* (as syn. *Collospermum microspermum*) and determined the remaining material on the specimen (K000524882) as *Astelia hastata* (as syn. *Collospermum hastatum*).

#### Excluded species.

*Astelia spicata* Colenso, Trans. & Proc. New Zealand Inst. 14: 335. 1882, *nomen illegitimum*. Type: NEW ZEALAND. North Island. In the forests about Kopua and Norsewood, *Colenso*. (Lectotype: K [000524878, digital image!], pistillate, designated by Skottsberg, 1934, 81). Moore (1966) regarded the type and other specimens examined to represent very small individuals of either *Astelia hastata* or *Astelia microsperma*.

*Collospermum spicatum* (Colenso) Skottsb., Kongl. Svenska Vetensk. Acad. Handl. Ser. 3, 14(2): 80. 1934; based on *Astelia spicata* Colenso *nomen illegitimum*.

*Astelia nana* Carse, Trans. & Proc. New Zealand Inst. 57: 91. 1926, *nomen illegitimum*. Type: NEW ZEALAND. North Island. Kaiaka (Mangonui County), Maungatapere (Whangarei County), Mauku (Franklin County), *H. Carse s.n.*; synonym of *Astelia spicata* Colenso *nomen illegitimum*. This is regarded by Skottsberg (1934) as a synonym of *Astelia spicata* Colenso *nomen illegitimum*. (Syntypes: CHR [328212, digital image!], staminate, pistillate; [328213, digital image!), pistillate; AK [3227!], pistillate; [3228!], pistillate; [222913!], staminate; [303282!], pistillate).

*Funckia spicata* Kuntze, Revis. Gen. Pl. 2: 711. 1891, *nomen rejiciendum*.

### Astelia montana

Taxon classificationPlantaeAsparagalesAsteliaceae

Seem.

Astelia montana Seem., Fl. Vit. [Seemann] 313, figs 1–6, pl. 95, 1865, non Reinecke 1898, nec Rechinger 1908. Type: FIJI ISLANDS. Kadavu, summit of Mbuke Levu mountain. *Seemann 641*. (Holotype: K [000524876, digital image!], pistillate; Isotypes: K [000524875, digital image!], vegetative; GH [00029835, digital image!], pistillate; BM [000990536, digital image!], pistillate).
Collospermum montanum (Seem.) Skottsb., Kongl. Svenska Vetensk. Acad. Handl. Ser. 3, 14(2): 73. 1934; based on *Astelia montana* Seem.
Funckia montana Kuntze, Revis. Gen. Pl. 2: 711. 1891, *nomen rejiciendum*.

#### Remarks.

Seemann stated in the preface of *Flora Vitiensis* (1965, iv) that “the first set of specimens collected by me were deposited at the Royal Herbarium, Kew and from these the plates accompanying this work have chiefly been taken”. The type material of *Astelia montana*, which was effectively published in *Flora Vitiensis*, can reasonably be expected to have been accessioned at Kew. Two sheets containing *Astelia montana* specimens collected by Seemann are accessioned at K. One sheet (K000524875) includes a vegetative plant collected at Mt. Mbuke Levu and a second sheet (K000524876) includes a leaf fragment annotated as collected at “Vuna, June 1860” and a pistillate inflorescence labeled as collected by Seemann (n. 641) in 1860. Sheet K000524876 includes the illustrations of the pistillate flower, staminode, berry and seed that appear in the plate that accompanies the protologue of *Astelia montana* (Seemann 1965).

The pistillate inflorescence labeled as Seemann’s collection n. 641 on sheet K000524876 represents the holotype. According to Seemann’s (1962, 1965) accounts of his field collections, he successfully ascended Mt. Mbuke Levu only once, on 6 September 1860. The vegetative specimen on sheet K000524875 was also collected by Seemann on “Buke Levu” [*sic*]. A type specimen can be mounted on multiple sheets “as long as the parts are clearly labeled as being part of that same specimen” (International Botanical Congress and JH Wiersema 2015); Article 8.3). Although it is likely that sheets K000524875 and K00524876 represent a single specimen that was mounted on separate sheets (Birch pers. comm., Smith 1979), they were not clearly labeled as such. Therefore, K000524875 is considered a duplicate. The leaf fragment on sheet K000524876 annotated as collected in “Vuna June 1860” may represent a fragment of an earlier collection from Vuna on the island of Taveuni (Smith 1979) where Seemann spent time during June 1860 (Seemann 1962).

### Astelia samoense

Taxon classificationPlantaeAsparagalesAsteliaceae

(Skottsb.) Birch comb. nov.

urn:lsid:ipni.org:names:77148140-1

Basionym: Collospermum samoënse Skottsb., Kongl. Svenska Vetenskapsakad. Handl. ser. 3, 14(2): 75. 1934. Syntypes: SAMOA ISLANDS. Upolo, 7 July, 1905, *F. Vaupel n. 356* (staminate); Aug. 1905, *K. & L. Rechinger n. 4334* (pistillate).
Astelia montana ex Reinecke, Die Flora der Samoa-Inseln. in Bot. Jahrb. Syst. vol. 25. 595. 1898; based on *Astelia montana* Seem.
Astelia montana ex Rechinger, Vegetationsbilder, series 6, issue 1. tafel 6. 1908; based on *Astelia montana* Seem.

#### Remarks.

Two specimens were identified by Skottsberg (1934) as types in the list of specimens examined. This included a staminate specimen (Vaupel 356 noted by Skottsberg (1934) as accessioned at HBG) and a pistillate specimen (K. and L. Rechinger n. 4334 noted by Skottsberg (1934) as accessioned at W). The pistillate specimen of Rechinger is not extant at W (pers. comm. A. Löckher, Department of Botany at Naturhistorisches Museum Wien). Efforts are underway to locate type material of *Astelia samoense* held in herbaria globally.

Diacritical signs, which should be suppressed in species names (International Botanical Congress and JH Wiersema 2015; Article 60.6) have not been transferred from *Collospermum samoënse*.

### Astelia subg. Tricella

Taxon classificationPlantaeAsparagalesAsteliaceae

Skottsb.

Astelia subg. Tricella Skottsb., Kongl. Svenska Vetenskapsakad. Handl. ser. 3, 14(2): 58. 1934.

#### Note.

Flowers with a short (Astelia sect. Periastelia) or long (Astelia sect. Tricella) perianth tube (0.3–3.5 mm); anthers dorsifixed, versatile; seeds ellipsoid, fusiform, or ovoid; funicle short, truncate, mucilaginous funicular hairs poorly developed; Type: *Astelia nervosa* Banks & Sol. ex Hook.f., designated here.

#### Remarks.

Astelia subg. Tricella is revised to include both Astelia sect. Tricella and Astelia sect. Periastelia, which form a well supported clade in phylogenetic analyses (Birch unpublished PhD thesis 2011, Birch et al. 2012). Astelia sect. Periastelia is monophyletic; however Astelia sect. Tricella is present as a grade and relationships within each of these sections remain equivocal.

### Astelia sect. Tricella

Taxon classificationPlantaeAsparagalesAsteliaceae

Skottsb.

Astelia sect. Tricella Skottsb., Kongl. Svenska Vetenskapsakad. Handl. ser. 3, 14(2): 58. 1934.

#### Note.

Compact to open growth form; leaves linear or ensiform; inflorescences bearing many flowers; staminate flowers with short or long filaments (0.8–3.5 mm); pistillate flowers with long outer tepals (2.3–5.3 × 1.2–2.6 mm); ovary trilocular, long (2.9–6.8 mm); fruit globose with few seeds per fruit (4–12); seeds long and wide (2.2–3.7 × 1.3–2.3 mm). Type: *Astelia nervosa* Banks & Sol. ex Hook.f., designated here.

#### Included taxa and distribution.

Australia: *Astelia australiana* (J.H.Willis) L.B.Moore, *Astelia psychrocharis* F.Muell. New Zealand: *Astelia chathamica* (Skottsb.) L.B.Moore, *Astelia fragrans* Colenso, *Astelia graminea* L.B.Moore, *Astelia grandis* Hook.f. ex Kirk, Astelia nivicola Cockayne ex Cheeseman var. nivicola, Astelia var. moriceae L.B.Moore, *Astelia nervosa* Banks & Sol. ex Hook.f., *Astelia petriei* Cockayne, *Astelia skottsbergii* L.B.Moore.

#### Habitat.

Lowland scrub (*Astelia chathamica*), temperate rainforest (*Astelia australiana*, *Astelia fragrans*, *Astelia grandis*, Astelia nivicola var. moriceae), sub-alpine and alpine herb-fields (*Astelia graminea*, *Astelia nervosa*, Astelia nivicola var. nivicola, *Astelia petriei*, *Astelia psychrocharis*, *Astelia skottsbergii*).

### Astelia sect. Periastelia

Taxon classificationPlantaeAsparagalesAsteliaceae

Skottsb.

Astelia sect. Periastelia Skottsb., Kongl. Svenska Vetenskapsakad. Handl. ser. 3, 14(2): 46. 1934.

#### Note.

Open growth form; leaves linear or ensiform; inflorescences bearing many flowers; staminate flowers with short or long filaments (0.9–2.8 mm); pistillate flowers with long outer tepals (2.3–5.3 × 1.2–2.6 mm); ovary trilocular, intermediate length (1.8–4.6 mm); fruit globose, few seeds per fruit (4–12); seeds long and narrow (1.8–3.6 × 0.7–1.6 mm). Type: *Astelia argyrocoma* A.Heller & Skottsb., designated here.

#### Included species and distribution.

Austral Islands, Rapa: *Astelia rapensis* Skottsb. Marquesas Islands, Ua Pou, Nuku Hiva: *Astelia tovii* F.Br. USA, Hawaii: *Astelia argyrocoma* A.Heller & Skottsb., *Astelia menziesiana* Sm., *Astelia waialealae* Wawra.

#### Habitat.

Lowland mesic forest (*Astelia menziesiana*, *Astelia rapensis*), tropical montane cloud forest (*Astelia argyrocoma* and *Astelia menziesiana*) and alpine swamps (*Astelia menziesiana* and *Astelia waialealae*).

### Incertae sedis

#### Astelia sect. Micrastelia

Taxon classificationPlantaeAsparagalesAsteliaceae

Skottsb.

Astelia sect. Micrastelia Skottsb., Kongl. Svenska Vetenskapsakad. Handl. ser. 3, 14(2): 56. 1934.

##### Note.

Low, compact, turf forming growth form; leaves caniculate; inflorescence bearing few flowers; staminate flowers with short filaments (0.6–1.9 mm); pistillate flowers with short outer tepals (2.6–3.8 × 0.8–1.3 mm); ovary trilocular, intermediate length (2.6–3.8 mm); fruit ellipsoid, many seeds per fruit (17–24); seeds short and narrow (1.1–1.7 × 0.5–0.8 mm). Type: *Astelia pumila* (Forst.) Gaudich.

##### Remarks.

The relationships of Astelia sect. Micrastelia are poorly resolved, with alternate relationships with Astelia subg. Asteliopsis and the clade containing Astelia subg. Tricella and *Collospermum* (Birch unpublished PhD thesis 2011, Birch et al. 2012). Astelia sect. Microastelia contains a single species, *Astelia pumila*, which is a compact, turf-forming plant and dominant component of *Astelia* moorland in Chile, the Falkland Islands, and Tierra del Fuego. As a cushion-forming species, it differs morphologically from Astelia subg. Asteliopsis, which contains species with open, spreading growth form that are epiphytic or terrestrial and primarily found of the understory in lowland to montane forests. *Astelia pumila* does share morphological features with Astelia subg. Asteliopsis (e.g. short pistillode or pistil) and, alternatively, with Astelia subg. Collospermum and Astelia subg. Tricella (e.g. seeds with a short, truncate funicle). The subgeneric placement of Astelia sect. Micrastelia remains equivocal and the section is unplaced (*incertae sedis*).

##### Included species and distribution.

Chile, Falkland Islands, Tierra del Fuego: *Astelia pumila* (Forst.) Gaudich.

##### Habitat.

Lowland (low latitudes) to sub-alpine (mid and higher latitudes) herbfields particularly on wet substrates (seeps, swamps etc.).

### Synoptic key

**Table:** 

1	Leaf tomentum with scales with stalk attached at base; anthers versatile; pollen densely echinate; ovary/pistillode with weakly to moderately-developed mucilaginous hairs that do not adhere to seed surface on drying	**2**
–	Leaf tomentum with peltate scales; anthers immobile; pollen sparsely spinulous; ovary/pistillode with well-developed mucilaginous hairs that adhere to seed surface on drying	**Astelia subg. Collospermum**
2	Ovary or pistillode unilocular, placentation parietal	**3**
–	Ovary or pistillode trilocular, placentation axile	**5**
3	Plants <40 cm tall; leaves generally less than 30 cm long; compact growth form including cushion and turf forming plants; inflorescence panicle (very reduced in *Astelia subulata*) < 7 cm long, 1–few flowered	**Astelia sect. Astelia**
–	Plants > 40 cm tall; leaves generally greater than 30 cm long; open growth form; inflorescence panicle > 9 cm long, many flowered	**4**
4	Leaves with acute apex; outer staminate tepals lanceolate, 5.0–6.4 mm long; pistillode bottle-shaped (lageniform); ovary ovoid; funicle long and curved	**Astelia sect. Palaeastelia**
–	Leaves with long acuminate apex; outer staminate tepals ovate, 2.5–5.0 mm long; pistillode ovoid-conical; ovary ampulliform or obpyriform; funicle short and ribbed	**Astelia sect. Desmoneuron**
5	Leaves generally < 10 cm long; compact turf forming growth form; inflorescence panicle < 7 cm long, 1-few flowered	**Astelia sect. Micrastelia**
–	Leaves generally > 10 cm long; compact or open growth form, but not turf forming; inflorescence panicle > 9 cm long, many flowered	**6**
6	Pistillode ampulliform, < 1.2 mm long; pistillate flowers with short outer tepals (2.7–4.0 × 0.7–2.0 mm); fruit ovoid, white, pink or maroon; seeds 1.3-2.2 mm long, 11–18 seeds per fruit; funicle ribbed	**Astelia sect. Isoneuron**
–	Pistillode ovoid, >1.0 mm, long; pistillate flowers with long outer tepals (2.3–5.3 × 1.2–2.6 mm); fruit globose, orange; seeds 2.0–3.6 mm long, 4–12 seeds per fruit; funicle not ribbed	**7**
7	Perianth tube 0.8-4.0 mm long; ovary 1.6–4.6 mm; seeds fusiform and narrow (0.7–1.6 cm)	**Astelia sect. Tricella**
–	Perianth tube 0.1-0.7 mm long, ovary 2.9–6.8 mm; seeds ovate and wide (1.3–2.3 cm)	**Astelia sect. Periastelia**

## Supplementary Material

XML Treatment for Astelia

XML Treatment for Astelia Banks & Sol. ex R.Br. subg. Astelia

XML Treatment for Astelia sect. Astelia

XML Treatment for Astelia sect. Palaeastelia

XML Treatment for Astelia subg. Asteliopsis

XML Treatment for Astelia sect. Desmoneuron

XML Treatment for Astelia sect. Isoneuron

XML Treatment for Astelia subg. Collospermum

XML Treatment for Astelia hastata

XML Treatment for Astelia microsperma

XML Treatment for Astelia montana

XML Treatment for Astelia samoense

XML Treatment for Astelia subg. Tricella

XML Treatment for Astelia sect. Tricella

XML Treatment for Astelia sect. Periastelia

XML Treatment for Astelia sect. Micrastelia

## Appendix 1

Specimens examined for generation of morphological data. Herbarium abbreviations follow Index Herbariorum (Thiers [continuously updated]). For each specimen, the following data are provided: sampled taxa, voucher specimen information [collection location, date, collector, collection number, herbarium, and herbarium accession number]

***Astelia argyrocoma*** A.Heller & Skottsb. - HAWAIIAN ISLANDS. Kauai: 10–16 Sept 1895, *A. A. Heller 2752* (BISH120976); Awaawaphui Trail, Honopu, 29 Dec 1956, *H. St. John 36004* (BISH413786); Kawaihau District, Lihue-Koloa Forest Reserve, Powerline trail from Wailua to Princeville, 27 Feb 1987, *T. Flynn 2063, R. Read, B. Read, D. Harder, and S. Weiss* (PTBG018401); Kawaihau District, north facing cliffs and forested slopes below Kekoiki, 9 Feb 1993, *K. R. Wood 2366, T. Flynn, D. Lorence, and S. Perlman* (PTBG014508); Na Pali-Kona Forest Reserve, Awaawapuhi Trail, 6 Dec 2007, *J. L. Birch 137, D. Lorence, K. Wood, and R. Aqurainja* (BISH751095); Na Pali-Kona Forest Reserve, Pihea Trail, 6 Dec 2007, *J. L. Birch 141, D. Lorence, K. Wood, and R. Aqurainja* (BISH751094); Koloa District, Lihue-Koloa Forest Reserve, Wahiawa Bog, 10 Apr 1987, *D. Lorence 5171, T. Flynn, R. DeLappe, W. H. Wagner, Jr. and F. Wagner* (PTBG018403); Koloa District, Lihue-Koloa Forest Reserve, Wahiawa Mountains, jeep road to microwave towers on Mt. Kahili, 2 Mar 1987, *D. Lorence 5105 and T. Flynn* (PTBG018404); Na Pali-Kona Forest Reserve, N. W. end of Alakai Swamp, 27 Dec 1930, *H. St. John 10768* (BISH120985); Pihea trail, on the flats above Lehua maka noe, 30 Jul 1963, *W. Takeuchi 140a* (BISH507424); Wahiawa Mts., Aug 1909, *C.N. Forbes 280K and J. M. Lydgate* (BISH120978); Waimea Drainage Basin, west side, 3-18 Jul 1917, *C.N. Forbes 849K* (BISH120969).

***Astelia alpina*** R.Br. **var. alpina** - AUSTRALIA. Tasmania: 1844, *M. Verreaux s.n.* (P00614764); Table Mountain, *R. Brown 5652* (K000524938); *R. Brown s.n.* (P00614768); Table Mountain, *R. Brown s.n.* (MEL727772); *R. Brown s.n.* (P00614766); *F. v. Mueller s.n.* (K000524934); Mt. Field National Park, Mt Field East, 1876, *F. v. Mueller s.n.* (K00524936); 24 Jan 1983, *S. J. Forbes 1277* (MEL1523734); 14 Feb 1989, *N. G. Walsh 2300* (MEL1577414); Mt. Field National Park, Mt. Rodway tow, 8 Jan 2009, *J. L. Birch 369* (MEL); Mt. Field National Park, Mt. Rodway tow, 8 Jan 2009, *J. L. Birch 370* (MEL); Mt. Field National Park, Uraquat Trail, 8 Jan 2009, *J. L. Birch 372* (SP094921); Mt. Field National Park, Uraquat Trail, 8 Jan 2009, *J. L. Birch 373* (CHR, BISH751060); Cradle Mountain-Lake St. Clair National Park, Marian’s Lookout trail, 11 Jan 2009, *J. L. Birch 379* (MEL); Southwest National Park, Mt. Eliza Plateau, 13 Jan 2009, *J. L. Birch 387 and A. Buchanan* (BISH751062); Tasmania: Southwest National Park, Mt. Eliza Plateau, 13 Jan 2009, *J. L. Birch 388A and A. Buchanan* (MEL); Southwest National Park, Mt. Eliza Plateau, 13 Jan 2009, *J. L. Birch 389 and A. M. Buchanan* (MEL); Southwest National Park, Mt. Eliza Plateau, 13 Jan 2009, *J. L. Birch 390 and A. M. Buchanan* (MEL).

**Astelia alpina var. novae-hollandiae** Skottsb. - AUSTRALIA. Victoria: Mt. Baw Baw, 1863, *B. F. v. Mueller s.n.* (P00614770); Mt. Baw Baw Alpine Resort, between summit t-bar and Painted Run t-bar, 18 Dec 2008, *J. L. Birch 343* (MEL); Mt. Baw Baw Alpine Resort, between summit t-bar and Painted Run t-bar, 18 Dec 2008, *J. L. Birch 344* (MEL); Mt. Baw Baw Alpine Resort, between summit t-bar and Painted Run t-bar, 18 Dec 2008, *J. L. Birch 345* (MEL); Mt. Baw Baw Alpine Resort, Village Trail, 18 Dec 2008, *J. L. Birch 346* (MEL); Mt. Buffalo National Park, Lyrebird Plains, 19 Dec 2008, *J. L. Birch 347* (MEL); Mt. Buffalo National Park, Bogong Plains, 19 Dec 2008, *J. L. Birch 348* (MEL); Mt. Buffalo National Park, Bogong Plains, 19 Dec 2008, *J. L. Birch 349* (MEL); Alpine National Park, Big River Fire trail, 20 Dec 2008, *J. L. Birch 350* (MEL); Alpine National Park, Big River Fire trail, 20 Dec 2008, *J. L. Birch 351* (BISH751063). New South Wales: Kosciuszko National Park, Kosciuszko summit trail, 30 Dec 2008, *J. L. Birch 357 and A. Beehag* (MEL).

***Astelia australiana*** (J.H.Willis) L.B.Moore - AUSTRALIA. Victoria: Beenak, 20 Dec 1970, *A. C. Beaglehole and B. A. Fuhrer ACB38542* (MEL534348); Beenak, 10 May 1984, *N. G. Walsh 1244* (MEL662242); Eastern Highlands, 11 Jul 1981, *N. G. Walsh s.n.* (MEL 599643‒44); Eastern Highlands, 18 Mar 1967, *J. H. Willis s.n.* (MEL224481‒3, MEL235432); Great Otway National Park, Brown Town Track, 22 Dec 2008, *J. L. Birch 352 and S. McDougall* (MEL); Otways, c. 150 m along Brown Town track from the Lavers Hill-Beech Forest road (just into State Forest on right hand side of Brown Town track), 6 Apr 1990, *R. Robinson s.n.* (MEL1581443‒44); Otway Range, Browntown Road, Weeaproinah (head of a tributary of Youngs Creek), 22 Nov 1990, *G. Beilby s.n.* (MEL227997‒8); Pioneer Creek, +/‒ 4 miles S.E. of Powelltown, 22 Nov 1969, *J. H. Willis s.n. and A. Morrison* (MEL224479); Yarra State Forest, 16 Dec 2008, *J. L. Birch 342 and* J. Downe (BISH751064).

***Astelia banksii*** A.Cunn. - NEW ZEALAND. *E. Cosson s.n.* (P00614762-3). Little Barrier Island: South of Parihakoakoa Stream, base of cliffs above shore, *R. Melville 6573 and E. H. Godley* (CHR129107). North Island: Auckland, cliffs at Takapuna Beach, Apr 1887, *D. Petrie s.n.* (SP084324); Auckland, *T. Kirk s.n.* (MEL5315); Auckland Ecological Region, Awhitu Ecological District, Matakawau, end of Hatton Road, sandstone cliffs bordering Manakau Harbour, 20 Apr 2001, *P. A. Aspin s.n.* (AK253724); Omahu Islets, Eastern Northland and Islands Ecological Region and District, south end Oakura Bay, top E. side of W. islet, 26 Apr 2003, *E. K. Cameron 11668* (AK280946, AK280988); Opotiki, Hukutaia Domain, 15 Jun 1962, *N. Potts s.n.* (CHR132070); Waitakere Ranges, Piha Beach, low cliff adjacent to trailhead to Laird Thomson trail, 16 Dec 2007, *J. L. Birch 228* (BISH751076); Waitemata, *T. Kirk s.n.* (SP084485). Poor Knights Islands: Tawhiti Rahi Island, Whangarei County, near campsite in Landing Bay, 22 Mar 1984, *A. E. Wright 6337* (AK166389, AK166423). Rakitu (Arid) Island: Hauraki Gulf, west of Te Akau Point, 6 Jan 1981, *E. K. Cameron A183* (AK268645).

***Astelia chathamica*** (Skottsb.) L.B.Moore - NEW ZEALAND. Chatham Islands: *J. D. Hector M.D. s.n.* (SP030867); Oct, *Travers 48* (MEL5298); F. v. Mueller (MEL5297); Chatham Island, *H. Travers s.n.* (SP034015A‒B); *H. N. Travers s.n.* (SP033784); Chatham Islands source (cultivated at 17 Holmwood Road, Christchurch), 8 Apr 1966, L. B. Moore s.n. (CHR150989A‒C); Chatham Islands source (cultivated at 17 Holmwood Road, Christchurch), 7 Nov 1967, L. B Moore s.n. (CHR181613A—D); *W. M. Martin s.n.* (SP080105A‒B); Chatham Islands source (G. W. Ramsay) (cultivated in Botany division garden), 29 Feb 1972, *L. B. Moore s.n.* (CHR233293A‒B). Chatham Island: 8 Sept 1966 (SP030867C,G,H); Tuku Valley, Timihunga, 19 Oct 1980, *A. M. Ringer s.n.* (AK170715). Pitt Island: Canister Cove, 5 Jan 1970, *N. C. Simpson s.n.* (SP0 42759).

***Astelia fragrans*** Colenso - NEW ZEALAND. North Island: Bay of Islands County, Puketi Forest, side ridge south of Bramley’s Ridge, north of Waikape Stream, 12 Oct 1984, *P. J. Bellingham s.n.* (AK170933); Kahikatea bush, Elsthorpe Scenic Reserve, Hawkes Bay (near Otane), 28 Oct 1963, *I. M. Morice 41* (CHR146207A‒B); Egmont National Park, Curtis Falls Track, 11 Dec 2007, *J. L. Birch 205* (AK334003); Smith’s Creek, Kaitoke, 19 Oct 1964, *L. B. Moore s.n.* (CHR141163); South Auckland Land District, Ikawhenua Ranges, Galatea, near Hikurangi Trig, 28 Dec 1993, *K. A. Ford 6/94* (CHR507187A‒B). South Island: Haast side of Grassy Creek (ca. 2 miles), 3 Dec 1962, *I. M. Morice s.n.* (CHR133152A‒C); Kahurangi National Park, Heaphy Track, 3 Jan 2008, *J. L. Birch 243 and K. E. Brown* (HAW); Karamea bluffs, Plot 2, 9 Jan 1985, *P. Wardle s.n.* (CHR419194A‒C); Kohaihai River (1 mile south of river); Murchison, 12 Oct 1965, *L. B. Moore and J. Clarke s.n.* (CHR159055); Pelorus River Bridge, Marlborough, 12 Oct 1965, *J. B. Irwin s.n.* (CHR159046A‒B), Resolution Bay, Marlborough Sounds, 18 Oct 1965, *J. B. Irwin s.n.* (CHR159046A‒B); Taumarunui Co., Scenic Reserve at Moerangi, 21 Nov 1981, *R. O. Gardner 3165* (AK158745); W. Nelson, 1 Jan 1964, *I. M. Morice 81* (CHR146986A‒E). Stewart Island: Northeastern Long Island, off Stewart Island, 8 Nov 1968, *J. Dugdale s.n.* (CHR188040).

***Astelia graminea*** L.B.Moore - NEW ZEALAND. South Island: E Nelson, Bryant Range, 5 km. S. W. of Mt. Duppa, Nov 1982, *A. P. Druce* (CHR393919); Mt. Richmond Forest Park, Dun Saddle, Maungatapu Track, 18 Jan 2008, *J. L. Birch 271* (BISH751067); Mt. Richmond Forest Park, Dun Saddle, Maungatapu Track, 18 Jan 2008, *J. L. Birch 272* (SP094949). NW Nelson, Boulder Lake Basin, near Darby Pond and Owen Creek, 10 Apr 1966, *I. M. Morice 327* (CHR170909); NW Nelson, Douglas Range, Jan 1966, *K. H. Marshall s.n.* (CHR141454); NW Nelson, Gouland Downs, (grown in shade house at Lincoln), 26 Apr 1968, *J. Clarke s.n.* (CHR182561); NW Nelson, Mt. Arthur summit trail, 15 Jan 2008, *J. L. Birch 257, S. Courtney, and R. Gaskill* (BISH751070); NW Nelson, Mt. Arthur summit trail, New Zealand, 16 Jan 2008, *J. L. Birch 259, S. Courtney, and R. Gaskill* (CHR); Nelson, Granity Pass, Mt. Owen, 11 Jan 1983, *K. H. Platt s.n.*(CHR520447); Nelson, Travers Range, Third Basin, 23 Apr 1962, *L. B. Moore s.n.* (CHR125634B); Nelson, Travers Range, Third Basin, 24 Apr 1962, *L. B. Moore s.n.* (CHR129010A-B).

***Astelia grandis*** Hook.f. ex Kirk - NEW ZEALAND. North Island: Dairy flat, Waitemata Co. 16 Dec 1963, *C. Earwaker s.n.* (CHR146924A-B); Junction of Puketaha Road/Gordontown Road, Hamilton, 14 Dec 2007, *J. L. Birch* 223 (BISH751075); Egmont National Park, Potaema Track, 11 Dec 2007, *J. L. Birch* 201A‒B (AK334001); North Cape, North Cape Scientific Reserve, Ngaroku Stream, 29 Dec 1986, *R. E. Beever 87003* (AK176615); Northland, Karikari, Tokerau Beach, Hector Busby property, 17 Oct 2006, *M. E. Young and L. J. Forster s.n.* (AK297667‒70); South Auckland province, Whanganui, 15 Oct 1895 (SP084426); W Hokio, west of Levin, Oct 1967, *A. P. Druce s.n.* (CHR179566); Waitomo Co., Waitanguru, 15 Feb 1967, *R. Bell and N. Potts s.n.* (CHR174847A-B). South Island: Lake Ianthe (cultivated at Lincoln), 10 Nov 1966, *J. Clarke s.n.* (cultivated) (CHR566424A‒B); Moeraki Valley, South Westland, 3 Apr 1978, *P. Wardle s.n.* (CHR321183A-C); Te Kinga near Lake Brunner, 14 Nov 1965, *N. Lambrechtsen s.n.* (CHR168155A‒C).

***Astelia hastata*** Colenso - NEW ZEALAND. 1875, *M. Filhol s.n.* (P00614792). North Island: Atuanui State Forest, 6 Apr 1969, *R. E and J. Beever 236* (CHR 195414A‒B); Auckland Ecological Region, Awhitu Ecological District, Pollok, S off Barthow Road, Craig’s Bush, lower part eastern end, 9 Jun 2005, *P. A. Aspin s.n.* (AK294016‒7); Auckland Ecological Region, Hunua Ecological District, Hunua Range, Mangatangi Kauri area, 4 Feb 1971, *I. L. Barton* (AK208895); Egmont National Park, Stony River, May 1961, *A. P. Druce s.n.* (CHR129729); Rotorua County, Lake Rotoehu, 29 Jan 1963, *R. Mason 100059* (CHR140253A‒C); South Manukau, Mauku, Jun 1901 (AK222913); Tainui Ecological Region, Kawhia Ecological District, Mount Pirongia, Ruapane, 13 Feb 1999, *P. J. de Lange 3801* (AK237952); Takaka, Rameka Gorge, 22 Feb 1964, *V. M. Scott s.n.* (CHR148397); Waikanae, Wellington, 5 Feb 1966, *I. M. Morice 312* (CHR150828A‒D); Wairoa, Hawkes Bay, Jan 1886, *A. Hamilton s.n.* (AK3191); Whangarei, Maungatapere, Aug 1989, *H. Carse s.n.* (AK3228). Poor Knights Islands: Motukapiti Island, (summit) Nature Reserve, 20 Nov 1984, *A. Penniket s.n.* (CHR418304A-B).

***Astelia hemichrysa*** (Lam.) Kunth - MASCARENE ISLANDS. Reunion Island: Forêt de Bébour, 26 Mar 1973, *D. H. Lorence* s.n. (MO2232208); Forêt de Bébour, 23 Feb 1979, *D. H. Lorence 2422* and *T. Cadet* (MO 2715190); Plateau de Belouve, 1956, *J. Bosser* 9462 (P00636603); Morne du Patates à Durand, 18 Mar 1974, *J. Bosser 21,643* (P00636604); *I. M. Morice P619* (CHR182114). Mauritius: Pétrin Nature Reserve, 3 Feb 1973, *D. Lorence M53* (MO222048); Pétrin Nature Reserve, 8 Oct 1978, *D. Lorence* 2100 (MO2715777).

***Astelia linearis*** Hook.f. **var. linearis** - NEW ZEALAND. South Island: NW Nelson, Lake Sylvester, 28 Mar 1964, *L. B. Moore s.n.* (CHR148475); Lake Sylvester above Cobb Dam, 9 Jan 1961, *P. Hynes 70325* (AK70325); Boulder Lake, Jan 1957, *A. E. Esler s.n.* (AK216795); Southland, Longwood Range, Bald Hill, 22 Jan 2008, *J. L. Birch 279 and P. Michel* (HAW); Southland, Longwood Range, Bald Hill, 22 Jan 2008, *J. L. Birch 281 and P. Michel* (CHR); Southland, Longwood Range, Bald Hill, 22 Jan 2008, *J. L. Birch 276 and P. Michel* (BISH751078); Westland, 3 Nov 1985, *P. N. Johnson 469* and *P. Wardle* (CHR420097); Denniston, near Westport, 5 Aug 1942, *I. A. McNeur 25407* (CHR25407); Port Pegasus, Stewart Island, *G. M. Thomson s.n.* (SP084464).

**Astelia linearis var. novae-zelandiae** Skottsb. - NEW ZEALAND. North Island: *W. Colenso 1587* (K000524926); Wellington Land District, Southern Ruahine Range, Mar 1971. *A. P. Druce s.n.* (CHR245761). South Island: Southland, Longwood Range, Bald Hill, 21 Jan 2008. *J. L. Birch 280 and P. Michel* (HAW); Fiordland National Park, Borland Saddle/Mt. Burns track, 24 Jan 2008, *J. L. Birch 290 and P. Michel* (MEL); Fiordland National Park, Borland Saddle/Mt. Burns track, 24 Jan 2008, *J. L. Birch 289 and P. Michel* (AK334015); Westland Land District, S. W. of Lewis Pass, 27 Dec 1962, *D. R. Given s.n.* (CHR142821); Wilberg Range, 27 Apr 1993, *P. Wardle s.n.* (CHR499828); Mt. Davy. *W. R. B. Oliver s.n.* (SP010664). Stewart Island: Port Pegasus, Bald Cove, Apr 1981. *I. M. Ritchie s.n.* (CHR372824); Rakiura National Park, Mt. Anglem, 26 Jan 2008. *J. L. Birch s.n.* (HAW); Rakiura National Park, Mt. Anglem summit, 26 Jan 2008, *J. L. Birch 300 and J. Blythal* (P02141677).

***Astelia menziesiana*** Sm. - HAWAIIAN ISLANDS. *J. F. Rock 8415* (BISH121100). Hawai‘i Island: ca. 0.5 miles east of National Park Road junction, 04 Aug 1964, *M. R. Crosby and W. R. Anderson 1972* (BISH121099); Hawaii Volcanoes National Park, Kilauea Research Center, 13 Aug 2009, *J. L. Birch 391* (HAW); Kau Forest Reserve, near boundary of Kapapala Forest Reserve, 1 Jul 1981, *J. Davis 560* (BISH657788); Kealakekua Ranch, S. Kona, 2 Apr 1980, *J. Davis 274* (BISH656028); Saddle Road, Kipuka No. 9. 27 July 1983, *T. Flynn 494* (PTBG019408); Olaa Forest Reserve, road from Hilo to Kulani Prison, 31 Oct 1950, *W. H. Hatheway 441* (BISH121085); South Kohala District, Umipoho Gulch area above Koia sanctuary, 16 Oct 1995, *K.R. Wood 4676* (PTBG033654); Waiakea mauka, July 1986, *W. Takeuchi and K. Shimabukuro 2699* (BISH503251); East Maui: Haleakala, upper Keanae valley, 19 Jul 1927, *O. Degener 4057* (BISH121037); Hana District, Hana Forest Reserve, above N rim of Kipahulu, 1974, *B. Harrison 395* (BISH429287); Hana District, E Haleakala, 2 mi N. E. of Paliku Cabin, N facing slope above Wai Anapanapa, *J. Henrickson and R. Vogl 3556* (BISH35823); Rainforest SW of Kaunuohua, 20 May 1982, *J. Davis 759* (BISH657637); Ukulele, 1919, *C. N. Forbes s.n.* (BISH121031); West Maui: 20 Jul 1964, *M. R. Crosby and W. R. Anderson 1864* (BISH121024); Puu Kukui Watershed Preserve, along boardwalk to summit at mile marker 2300, 31 Oct 2007, *J. L. Birch 154, R. Bartlett, D. Cole, L. Dunn, and D. Tanaka* (HAW); Puu Kukui Watershed Preserve, forest below bog, 31 Oct 2007, *J.L. Birch 158, R. Bartlett, D. Cole, L. Dunn, and D. Tanaka* (MEL); Puu Kukui Watershed Preserve, forest below bog, 31 Oct 2007, *J.L. Birch 159, R. Bartlett, D. Cole, L. Dunn, and D. Tanaka* (HAW); Molokai: Waikolu Valley, head of valley, 15 Mar 1952, *O. Degener 22160 and C. Tousley* (BISH10425); between Waikolu Valley and N. Puu Alii, 10 Apr 1928, *O. Degener 4065* (BISH121013); Pepeopae Bog, 25 m after end of boardwalk through bog, 22 Jun 2007, *J. L. Birch 149 and C. W. Morden* (BISH751101); ridge E of Mapulehu Valley, 29 Dec 1932, *H. St. John 12840* and F. Fosberg (BISH121017); Kawele, ridge to Pelekunu Pali, 17 Mar 1910, *J. F. Rock 6095* (BISH121053); Oahu. Waianae Range: Mt. Kaala, 14 Sept 2007, J. L. Birch 179 (HAW); Mt. Kaala summit trail (Waianae access), 26 Sept 2009, *J. L. Birch 393* (HAW); Mt. Kaala summit, 24 Nov 1929, *H. St. John 10071* (BISH121055); Kaukawahua gulch, N. fork, 15 May 1909, *J. F. Rock 4005* (BISH121110); Pukaloa gulch, above Schofield, off ridge trail to Puu Kalena, near Kumakalii, 16 Apr 1987, *S. Perlman 5640 and B. Hill* (BISH617222); Puu Hapapa, northeast ridge, 7 May 1939, *O. Degener 12382* (AK71043); Puu Hapapa, 16 Mar 1930, *E. Christopherson 1288B* (BISH121111); Puu Hapapa, 16 Mar 1930, *E. Christopherson 1288A* (BISH121113); Ridge between Puu Kanehoa and Puu Kaua, 23 Jun 1940, *O. Degener 12966* (BISH121146). Koolau Range: Kuliouou Valley, summit, 23 Jun 1935, O. Degener 10471, K. Park, and D. Topping (BISH121114); N Kaaawa, 12 Apr 1931, *E. P. Hume 189* (BISH121171).

***Astelia microsperma*** Colenso - NEW ZEALAND. North Island: Auckland Ecological Region, Rodney Ecological District, Mt. Tamahunga, 28 Feb 1993, *M. E. Young s.n.* (AK212022); Blue Mountains, near Pinehaven, Hutt Valley, 1 Jan 1963, *I. M. Morice s.n.* (CHR132056A‒C); Kaimai Mamaku Forest Park, Mt. Te Aroha, 13 Dec 2007, *J. L. Birch 248, C. Gemmill, E. Grove, and N. Wakefield* (SP094950); Mangonui County, Maungataniwha Range, Feb 1908, *H. Carse 516/1* (CHR328214 A‒B); Mt. Egmont, near Puniho Hut, 20 Jan 1963, *I. M. Morice s.n.* (CHR132058); Ohakune, Mt. Road, 13‒14 Dec 1962, *J. M. Wheeler s.n.* (CHR141652); Originally from Ruahine Range, west Tamaki River (cultivated in shade house at Lincoln), 2 Jan 1968, *J. Clarke s.n.* (CHR182141); Pureora State Forest Park, West Taupo, 26 Jan 1982, *J. E. Braggins 97* (AK270463); Tararua Forest Park, trail to Mt. Holdsworth, 12 Jan 2008, *J. L. Birch 244 and S. J. Birch* (BISH751080); Tongariro National Park, road between Ohakune and Turoa, 17 Mar 1999, *L. Perrie and L. Sheppard s.n.* (SP083500).

***Astelia montana*** Seem. - VANUATU ISLANDS. Aneityum: crête du Nezwon Netounemla, 21 Jul 1971, *J. Raynal and M. Schmid RSNH 16129* (CHR 298987; P00636616); Mt. Inréro, peak south of mountain, 21 Jul 1971, *P. S. Green in RSNH 1149* (P00636643). Espiritu Santo: Mt. Tabwemasana, 17 Aug 1985, *P. Cabalion 2816* (P000636609); Mt Tabwemasana, 2‒8 Sept 1971, *C. Wee-Lek RSNH 249* (P00636644). Tanna: Mt. Toukosméreu, 29 Jul 1971, *P. S. Green 1244* (P00636642). FIJIAN ISLANDS. Viti Levu: Water reserve near Suva, Jul 1965, *J. W. Dawson s.n.* (CHR170581, SP85966A‒B). Vanua Levu: Thakaundrove, Natewa Peninsula, Uluingala, 15 Jun 1934, *A. C. Smith 1978* (P00636610). Kandavu: Mt. Mbuke Levu summit, 6 Sep 1860, *B. Seemann 641* (GH00029835).

***Astelia nadeaudii*** Drake & F.Br. - SOCIETY ISLANDS. Tahiti: Mt. Aorai, 27 Apr 1858, *J. Nadeaud 250* (P0636625-28); Mt. Aorai, trail to summit from Fare Rau Ape, above Papeete on ridge east of Fautaua Valley, slopes of Rocher du Diable, 30 Mar 1973, *F. R. Fosberg 54,701 and M.-H. Sachet* (US2680616, US2680617); Mt. Aorai, 14 Dec 2006, *J. Meyers s.n*. (BISH751087); Mt. Aorai, sentier de l’Aorai, entre Fare Ata et le sommet, 15 Feb 1983, *J. Florence 4545* (P00636605); Mt. Marau, sentier du Pic. Vert., 28 Jan 1982, *J. Florence 2307* (P00636617, US3186926); Mt. Orohena, crête oust de la Papenoo, senteir de l’Orohena, 20 Oct 1982, *J. Florence 3992* (P00636618); Tahiti, 1847, *M. J. Lépine s.n.* (P00636635); Tahiti, *J. Nadeaud 250* (P00636622-24). Raiatea: Mt. Toomaru, Tevaitoa, crête sommitale N. du Mt. Toomaru, 27 Nov 1987, *J. Florence 8936* (P00636640).

***Astelia neocaledonica*** Schltr. - NEW CALEDONIA. 1868-1870, *M. Balansa 950* (P00636652); 19 Apr 1910, *M and Mme Le Rat 2874* (P00636651); *I. Franc 3125* (MO977220, MEL600656). Grande Terre: Forêt de Sailles, 5 Dec 2001, *J. Muzinger 1244, B. Suprin and F. Carriconde* (MO5839966); Mt. Koghi, Mar 1929, *M. Franc 2325* (P00636650); Mt. Mandjélia, 24 May 1980, *J. W. Dawson s.n.* (SP085969); Mt. Mandjélia, below radio tower ca. 5 air-km of Pouébo, 11 Apr 1980, *G. McPherson 2531* (PTBG023027-8); Mt. Mandjélia, below radio tower ca. 5 air-km of Pouébo, 11 Apr 1980, *G. McPherson 2532* (MO2922540); Mt. Mau, crête sommitale, 21 Aug 1940, *M. R. Virot 284* (P0636648); Province Nord, Mont Görö Até, 19 Nov 2002, *F. Tronchet 469 and J. Munzinger, D. and I. Létocart, J.-P. Butin, A. Oddi, and A. Obry* (MO4781294); Province du Nord, upper Amoa River Valley, trail from Alain Obry property to Görö Até, 23 Apr 2002, *I. Létocart 5644, P. Lowry II, G. D. McPherson, F. Carriconde, and D. Létocart* (MO5666374).

***Astelia nervosa*** Banks & Sol. ex Hook.f. - NEW ZEALAND. North Island: Coromandel County, Moehau summit, 2 Nov 1980, *R. O. Gardner 2763* (AK153040); Kokianga Co., Waima Forest, ridge between summit of Mount Misery and “highest point in Northland”, 16 Jan 1990, *A. E. Wright 9684* (CHR192727); Egmont National Park, Ski field Road, 11 Dec 2007, *J. L. Birch 207* (CHR); Kaimai Mamaku Forest Park, Mt. Te Aroha, Waikato, 13 Dec 2007, *J. L. Birch 220*, *C. Gemmill, E. Grove, and N. Wakefield* (SP094947); Otorohanga County, Ranginui summit, Rangitoto Range, 16 Dec 1981, *R. O. Gardner 3209* (AK158716); Table Mountain, Kauaeranga Valley, Coromandel Range, 10 Apr 1971, *I. M. Morice 484B* (CHR208245); Thames County, Lookout rocks, inland from Taran, 10 Dec 1986, *R. O Gardner 5049* (CHR484212); Tongariro (grown in cultivation at Lincoln), 10 Nov 1966, *J. Clarke s.n.* (CHR566443A‒B); Waitemata County, Albany Scenic Reserve, valley lying east of Lonely Track Road, Wright’s Road intersection, 6 Oct 1979, *R. O. Gardner s.n.* (AK150794); Wellington District, Tongariro National Park, ca. 1.5 km N. W. of Chateau Tongariro, nr. road to Chateau, Whakapapanui Track, 24 Mar 1970, *P. J. Edwards 74* (AK129710). South Island: Marlborough Land District, Mt. Stokes, Marlborough Sounds, Mar 1977, *A.P. Druce s.n.* (CHR310138); Mt. Stokes, *T. Kirk s.n.* (SP030865); Mt. Stokes Scenic Reserve, Mt. Stokes summit, Marlborough Sounds, 14 Jan 2008, *J. L. Birch 251 and J. Little* (BISH751065); Mt. Stokes Scenic Reserve, Mt. Stokes summit, Marlborough Sounds, 14 Jan 2008, *J. L. Birch 252 and J. Little* (CHR); Nelson Land District, Nelson, Aniseed Valley Scenic Reserve, 1972, *G. C. and D. Kelly s.n.* (CHR230190). Arthur’s Pass, Bealey Track near Margaret’s Tarn, 29 Nov 1961, *R. Melville 5478* (CHR129127, AK156821); Kahurangi National Park, Heaphy Track, 31 Dec 2007, *J. L. Birch 237 and K. E. Brown* (HAW); Kahurangi National Park, Heaphy Track, 2 Jan 2008, *J. L. Birch 240 and K. E. Brown* (SP094946A/B); Kahurangi National Park, Heaphy Track, James Mackay Hutt, *2 Jan 2008, J. L. Birch 241 and K. E. Brown* (P02141675); Kahurangi National Park, Heaphy Track, trail to Gouland Downs Hut, ca. 1 mile from Percy Hut, 31 Dec 2007, *J. L. Birch 238 and K. E. Brown* (SP094945); NW Nelson, Mt. Arthur, 16 Jan 2008, *J. L. Birch 263* (BISH751073); Mt. Arthur, 16 Jan 2008, *J. L. Birch 267* (CHR); NW Nelson, Boulder Lake, Orater Creek, 11 Apr 1966, I. M. Morice 344 (CHR170919); Cobb Valley, Lake Sylvester, just above forest hut, 31 Mar 1964, *L. B. Moore s.n.* (CHR148498A‒B); trail to Mt. Arthur, 15 Jan 2008, *J. L. Birch 258, S. Courtney, and R. Gaskill* (SP094941); Mt. Arthur trail to Gordon’s Pyramid, 16 Jan 2008, *J. L. Birch 260* (BISH751068); Takaka Hill, on road to Canaan, 11 Jan 1964, *I. M. Morice 106* (CHR146995); Nelson Lakes National Park, Ridge to Mole Tops, 14 Feb 1964, *M. J. A. Simpson 4115* (CHR148394); North Canterbury, Upper Clarence Valley, Mt. St. Patrick, 14 Jan 1972 (CHR 228672); Canterbury Land District, Deer Spur Walk, Peel Forest Park, Stop 5, 29 Oct 1970, *B. P. J. Molloy* (CHR212134); West Coast, Paparoa Range, Croesus track, 19 Jan 2008, *J. L. Birch 274* (AK334012); Oparara Arch, 22 Jan 1985, *P. Wardle s.n.* (CHR574281); St. Arnaud Range, 11 Dec 1950, *W. R. B. Oliver s.n.* (SP010631). Fiordland, Dec 1966, *P. K. Dorizac s.n.* (CHR174065); Fiordland, Mt. Gray, 14 Feb 1959, *M. J. A. Simpson 1154* (CHR111751); Fiordland, Secretary Island, Feb 1967, *P. Wardle (3)* (CHR 566430); Fiordland National Park, Hollyford Valley, 31 Jul 1965, *K. Dorizac s.n.* (CHR141436); Homer, Dec 1943, *J. Salmon s.n. with R. Forster* (SP084347); Milford Sound, Sinbad Gulley, 27 Feb 1975, *P. N. Johnson s.n.* (CHR 261738); Pigeon Saddle, Oct 1957, *A. Esler s.n.* (SP085190); Track from Key Summit to road, 16 Dec 1962, *I. Morice s.n.* (CHR133242); Track from Key Summit to road, 16 Dec 1962, *I. Morice s.n.* (CHR133243).

***Astelia nivicola*** Cockayne ex Cheeseman **var. nivicola** - NEW ZEALAND. South Island: Fiordland, Hunter Mts., east of summit of unnamed peak north of Green Lake, 9 Jan 1967, *D. Given 69043* (CHR193928); Fiordland, Key Summit, between upper bog and track upwards, *P. K. Dorizac (=IMM 474/2)* (CHR191766A‒B); Fiordland National Park, Borland Saddle/Mt. Burns track, 24 Jan 2008, *J. L. Birch s.n. and P. Michel* (HAW); Marlborough, Richmond Range, Mt. Richmond, 23 Nov 1961, *J. I. Townsend s.n.* (CHR366690); Mt. Ollivier, Sealey Range, 12 Feb 1919, *L. Cockayne 1272* (AK3224); NW Nelson, Mt. Centre, Jan 1977, *A. P. Druce s.n.* (CHR310405, CHR310406); Nelson Land District, NW Nelson, Mt. Centre, Jan 1977, *A. P. Druce s.n.* (CHR310406); Southland, Longwood Range, Bald Hill, 21 Jan 2008, *J. L. Birch 277 and P. Michel* (CHR).

**Astelia nivicola var. moriceae** L.B.Moore - NEW ZEALAND. South Island: Collected from Wilmot Pass, (grown in Bot. Div. shadehouse), 5 May 1970, *L. B. Moore s.n.* (CHR199865); Fiordland, near Henry Saddle on George Sound track, 16 Jan 1966, *P. K. Dorizac s.n.* (CHR150752, CHR150753); Nelson, Flora track, head of Pearse River (type locality), 16 July 1964, *L. B. Moore s.n.* (CHR 151212); Nelson, Flora track, halfway between Flora saddle and Flora Hut, 26 Dec 1964, *D. R. Given 64410* (CHR144190A‒B); Paparoa Range, above Roa Mine, 18 Dec 1965, *L. B. Moore, s.n., J. Clarke, and I. Robins* (CHR168145A‒C); On E side 15 minutes from Wilmot Pass, 14 Dec 1962, *I. Morice s.n.* (CHR133164A‒C); Track to Boulder Lake, The Pulpit, 6 Jan 1964, *I. M. Morice 92* (CHR146984A-C); Nelson, track to Mt. Arthur, 13 Apr 1963, *I. M. Morice* 12 (CHR 144249).

***Astelia papuana*** Skottsb. - PAPUA NEW GUINEA. Central Highlands, Mt. Wilhelm, 25 Aug 2011, *W. R. and M. N. Philipson 3493* (CHR198540), Eastern Highlands province, Kainantu sub-province, Mt. Piora, 9 Jan 1975, *M. J. S. Sands 1604, G. A. Pattison, and J. J. Wood* (US3248428); Goilala sub-district, Mt. Dickson, 11 Feb 1964, *T. G. Hartley* 12985 (US3485933); Mt. Wilhelm, Lake Aunde, 22-26 Sep 1962, *F.W. Went 223* (MO1806059); South Highland district, Tari sub-district, Mt. Ambua, 29 July 1966, *W. Vink 17287* (MO2322009); Western Highlands district, Hagen sub-district, Mt. Hagen, 2 July 1967, *J. M. Wheeler ANU 6385* (US3321035); West Highlands, Mt. Kegum, 8 Apr 1977, *J. F. Veldkam 7598 and A. Vinas* (MO2682556); West Sepik district, Telefomin sub-district, Star Mountains, Mt. Scorpion, 25 May 1975, *J.R. Croft and G.S. Hope LAE 68018* (US2895090); West Sepik district, Telefomin sub-district, Mt. Capella north of east summit, 18 Apr 1975, *W. R. Barker LAE 67438 and T. Umba* (MO3270313).

***Astelia petriei*** Cockayne - NEW ZEALAND. South Island: Arthur’s Pass, Temple Basin, 8 Dec 1963, *B. H. MacMillan 3* (CHR146933); Fiordland National Park, Gertrude Saddle track, 23 Jan 2008, *J. L. Birch 287 and P. Michel* (SP094956); Fiordland National Park, Gertrude Saddle track, 23 Jan 2008, *J. L. Birch 286 and P. Michel* (CHR); Fiordland National Park, Borland Saddle/Mt. Burns track, 24 Jan 2008, *J. L. Birch 291 and P. Michel* (SP094952); Molesworth Ecological Region, Balaclava Ecological District, Island Saddle just north of road summit, 4 Jan 2002, *E. K. Cameron 10684* (AK255636); N. Canterbury, Upper Clarence Valley, Mt. St. Patrick, 14 Jan 1972 (CHR228674); NW Nelson, Mt. Arthur, 15 Jan 2008, *J. L. Birch* 254, *S. Courtney, and R. Gaskill* (BISH751072); NW Nelson, Mt. Arthur, 15 Jan 2008, *J. L. Birch 255*, *S. Courtney, and R. Gaskill* (MEL); Otago Province, Lake Harris Saddle (cultivated in Dunedin), 1923, *W. A. Thomson s.n.* (SP085977); Westland National Park, Fox Glacier, Chancellor Hut, 2 Feb 1973, *P. Wardle s.n.* (CHR218793A‒B).

***Astelia psychrocharis*** F.Muell. - AUSTRALIA. New South Wales: Mt. Kosciuszko, 19 Feb 1990, *M. G. Corrick 10668* (MEL1578958); Mt. Kosciuszko, June 1901, *C. H. Grove s.n.* (MEL 2213620); Kosciuszko National Park, Mt. Kosciuszko, 30 Dec 2008, *J. L. Birch 359 and A. Beehag* (MEL); Kosciuszko National Park, Mt. Kosciuszko, 30 Dec 2008, *J. L. Birch* 360 *and A. Beehag* (MEL); Kosciuszko National Park, Mt. Kosciuszko, 31 Dec 2008, *J. L. Birch 361 and A. Beehag* (MEL); Kosciuszko National Park, Mt. Kosciuszko, 31 Dec 2008, *J. L. Birch 362 and A. Beehag* (BISH751056); Kosciuszko National Park, Southern Tablelands, near end of Mt. Blue Cow, “Snowtube”, 31 Jan 1990, *J. H. Willis s.n.* (MEL 2117076).

***Astelia pumila*** (Forst.) Gaudich. - ARGENTINA. Tierra del Fuego: Isla de los Estados, Bahia San Antonio, Puerto Hoppner, 8 Nov 1971, *T. R. Dudley, R. N. P. Goodall, and G. Crow 1596* (MO2300439); Northwest side of Bahia Thetis, 14 Nov 1969, *R. N. P. Goodall 2248* (US2626023). CHILE. Chiloé Island: River Toigoi, Chepu, 26 Oct 1958, *E. J. Godley 349a* (CHR 547077B); River Toigoi, Chepu, 26 Oct 1958, *E. J. Godley 348* (CHR 55303). Magallenes y de la Antártica Chilena: Churucca, 30 Jan 1879, *Lud Savatier 189* (P00614743); Détroit de Magellan-Port Famine. *M. le Guiillou 188* (P00614753). Riesco Island: Peninsula Cordova, Pto. Condor, 29 Aug 1970, *E. Pisano V. 2639* (MO2384477). FALKLAND ISLANDS. [Iles Maclovian] 1825, *Gaudichaud s.n.* (P00614740); [Iles Maclovianl 1825, *Gaudichaud s.n.* (P00614741).

***Astelia rapensis*** Skottsb. - AUSTRAL ISLANDS. Rapa: Kaimaru, south ridge of Mt. Perahu, 13 Jul 1934, *H. St. John and J. Maireau 15,513* (BISH509590, P00636647).

***Astelia samoense*** (Skottsb.) Birch - SAMOAN ISLANDS. Savai‘i: Cloud forest near Mt. Silisili, 17 June 1992, *A. Whistler 8861* (housed at HAW); Mata-ole-afi cinder cone, 29 May 1975, *A. Whistler W2492* (housed at HAW); Matavanu Crater, 24 July 1931, *E. Christophersen and E. Hume 2130* (MO1631444); Mt. Silisili, 30 May 1975, *A. Whistler W2518* (housed at HAW). Upolu: 22 Nov 1973, *A. Whistler AW1172* (housed at HAW); between Mt. Lepu‘e and Mt. Fito, 15 Oct 2008, *A. Whistler 12035* (housed at HAW); Lake Lano, 6 Aug 1971, *A. Whistler W270* (housed at HAW); Laka Lanoto‘o, 15 Aug 1970, *A. Whistler W2177* (housed at HAW); Mt. Siga‘ele, 19 July 1973, *A. Whistler W358* (housed at HAW); Mt. Lepu‘e east of Tiavi, 7 Dec 1973, *A. Whistler W1251* (housed at HAW).

***Astelia skottsbergii*** L.B.Moore - NEW ZEALAND. South Island: Douglas Range, 15 May 1963, *K. H. Marshall s.n.* (CHR146875A‒D); NW Nelson, Boulder Lake, Brown Cow Saddle, 12 Apr 1966, *I. M. Morice 350* (CHR170925); NW Nelson, Douglas Range, 1 Jun 1964, *K. Marshall s.n.* (CHR151057); NW Nelson, Mt. Domett, Jan 1977, *A. P. Druce s.n.* (CHR311624); NW Nelson, Mt. Goul, 19 Jan 1973, *M. J. A. Simpson 7215* (CHR278243); Mt. Arthur Plateau, Gordon’s Pyramid, 26 Feb 1965, *R. Macfarlane s.n.* (CHR141321); Kahurangi National Park, Mt. Arthur, 16 Jan 2008, *J. L. Birch 261* (BISH751069); Kahurangi National Park, Mt. Arthur, 16 Jan 2008, *J. L. Birch 262* (CHR); Waingaro Peak, where Douglas and Lockett Ranges meet, on North facing slope, 16 Jan 1970, *I. M. Morice s.n.* (CHR199791); Wangapeka, Mt. Luna, 23 Jan 1971, *I. M. Moore s.n.* (CHR215917A‒B).

***Astelia solandri*** A.Cunn. - NEW ZEALAND. Little Barrier Island: North Auckland Land District, 24 Oct 1965, *R. Melville 6672* (CHR129112). North Island: Anatoki Valley, 9 Jan 1964, *I. M. Morice 100* (CHR147598); Auckland, 1875, *M. Filhol s.n.* (P00614785); Auckland Ecological Region, Awhitu Ecological District, Matakawau Reserve, south of Matakawau Road, 23 Apr 2001, *P. A. Aspin s.n.* (AK253912, AK254486); Coromandel, Dec 1905, *D. Petrie s.n.* (SP85958); Egmont Ecological Region and District, Mt. Egmont, Aug 1958, *A. E. Esler s.n.* (AK219600); Hunua, Dec 1868, *T. Kirk s.n.* (SP84518); Hunua, Dec 1868, *T. Kirk s.n.* (SP084519); Kaimai Mamaku Forest Park, Mt. Te Aroha summit trail, 13 Dec 2007, *J. L. Birch 219, C. Gemmill, E. Grove, and N. Wakefield* (SP094962); Mauku, Franklin County, 5 May 1901, *H. Carse s.n.* (CHR328230); Egmont National Park, Potaema Track, 11 Dec 2007, *J. L. Birch 202* (SP094922); Egmont National Park, road below Dawson Falls, 20 Jan 1964, *I. M. Morice 119* (CHR148342); Mt. Wellington lava fields near Auckland, Apr 1897, *D. Petrie s.n.* (SP084507); Mt. Wellington, Auckland, 4 Apr 1922, *H. Carse s.n.* (CHR292211); North Tararua Range, Makotukutuku St. near Mt. Kaiparoro, Jan 1977, *C. Ogle s.n.* (CHR286974); Orotere, south of Kaeo, 26 Aug 1965, *R. Cooper s.n.* (AK118465); Tararua Forest Park, Mt. Holdsworth, 12 Jan 2008, *J. L. Birch 250 and S. J. Birch* (BISH751081). South Island: Nelson (SP08453). Poor Knights Islands: Whangarei County, Aorangi Island, 31 Aug 1984, *A. E. Wright 6564* (AK169414). Rangitoto Island: North Auckland Land District, 13 Feb 1965, *I. M. Morice s.n.* (CHR 566383).

***Astelia subulata*** Cheeseman - NEW ZEALAND. Campbell Island: Homestead Ridge, 26 Dec 1998, *C. D. Meurk s.n.* (CHR537469); Tucker Cove, 14 Jan 1947, *W. B. Brockie* s.n. (CHR223725). South Island: Paparoa Range, Westland, 14 Jan 1967, *L. B. Moore s.n and J. Clarke* (CHR174718). Stewart Island: Table Hill, 12 Jan 1940, *L. B. Moore* s.n. (CHR24184); Rakiura National Park, Mt. Anglem, 26 Jan 2008, *J. L. Birch s.n. and J. Blythal*, (HAW); Rakiura National Park, Mt. Anglem, 26 Jan 2008, *J. L. Birch 299 and J. Blythal*, (CHR); Rakiura National Park, Mt. Anglem, 26 Jan 2008, *J. L. Birch 301 and J. Blythal* (BISH751079).

***Astelia tovii*** F.Br. - MARQUESAS ISLANDS. Nuku Hiva: Summit of the ridge south of Tekao, between the new airport road at peak #1227 M. and Tekao, the main ridge above Toovii, 25 Sept 1995, *S. Perlman 15,055* (BISH660310, MO5601362, PTBG021995); Mt. Tapuaooa, about 2 km. from the Tapuaooa shelter and 3 km. W of Mt. Ooumu, 10 Jul 1970, *G. W. Gillett 2166* (US 3485393, P00636646); Toovii, épaulement SE du Mt. Tekao, 28 May 1984, *J. Florence 6818* (P00636614); Tovii, ridge above L’Economie Rurale to Ooumu, 17 Jul 1988, *W. L. Wagner, D. Lorence, J. Florence, and S. Perlman 6119* (MO4330721, PTBG008791, P00636608, US3206556); Toovii Plateau, trail behind L’Économie Rurale toward Ooumu peak, near summit crest, 16 Jul 1988, *S. Perlman 10,127* (PTBG008286).

***Astelia trinervia*** Kirk - NEW ZEALAND. North Island: Auckland Ecological Region, Awhitu Ecological District, Matakawau Reserve, south of Matakawau Road, 23 Apr 2001, *P. A. Aspin s.n.* (AK253912); Eastern Northland Ecological Region, Eastern Northland & Islands Ecological District, Orotere, south of Kaeo, 26 Aug 1965, *R. C. Cooper s.n.* (AK118465); North Auckland Land District, Franklin Co., Mauku, 5 May 1901, *H. Carse s.n.* (CHR328230); North Auckland, Hunua, Wairoa [Hunua] Falls, Dec 1868, *T. Kirk s.n.* (SP084519); North Auckland Land District, Mt. Wellington, Auckland, lava field, 4 Apr 1922, *H. Carse s.n.* (CHR292211); North Auckland, Auckland City, Mt. Wellington lavafield, Apr 1897, *D. Petrie s.n.* (SP084507). Little Barrier Island: North Auckland Land District, summit, 9 Mar 1962, *R. Melville 6672* (CHR129112). Rangitoto Island: North Auckland Land District, 13 Feb 1965, *I. M. Morice s.n.* (CHR 566383).

***Astelia waialealae*** Wawra - HAWAIIAN ISLANDS. Kaua‘i: Alakai Swamp, transect across Sincock Bog, Bog 1, 13 Feb 1989, *S. Perlman 10,641, T. Pratt, and J. Lepson* (PTBG011706); Alakai Swamp, 1 Sep 1977, *P. van Royen 11716 and J. Davis, S. Perlman* (BISH419748A); Waialealae, 23 Sep 1909, *J. F. Rock 5041* (BISH121147); Waialealae, 20 Oct 1911, *J. F. Rock 8878* (BISH121150); Waimea District, Sincock Bog, Halehaha area, *S. Perlman and K. Wood 3395* (PTBG019596).
