# Corrigenda: A revision of infrageneric classification in *Astelia* Banks & Sol. ex R.Br. (Asteliaceae)

**DOI:** 10.3897/phytokeys.56.6517

**Published:** 2015-10-09

**Authors:** Joanne L. Birch

**Affiliations:** 1 Department of Botany, University of Hawaii at Manoa, Honolulu, HI, 96822, USA; 2 Present address: Royal Botanic Gardens Victoria, Birdwood Avenue, Melbourne, Victoria 3004, Australia

In Birch (2015), *Astelia banksii* A.Cunn. was incorrectly stated as present in New Caledonia within the “Included species and distribution” section for Astelia sect. Isoneuron Skottsb. (page 112). The correct distribution of *Astelia banksii* A.Cunn. is New Zealand. The Astelia sect. Isoneuron Skottsb. “Included species and distribution” section should read: Included species and distribution. New Caledonia: *Astelia neocaledonica* Schltr. New Zealand: *Astelia banksii* A.Cunn.

Additionally, the designation of the *Astelia microsperma* Colenso *pro parte* type was incorrectly stated (page 113) as being made by Skottsberg on page 81 (1934). This type designation was made by Skottsberg on page 87 (1934). The designation of the *Astelia microsperma* type should correctly read:

*Astelia microsperma* Colenso *pro parte*, Trans. & Proc. New Zealand Inst. 17: 251. 1885 (description of fruit only). Type: NEW ZEALAND. North Island. Seventy-mile Bush, between Norsewood and Dannevirke, County of Waipawa. 1884, *W. Colenso s.n.* (Lectotype: K [000524879, digital image!], fruiting material in packet, designated by Skottsberg, 1934, 87).
